# Explanation for the signs and symptoms of tooth eruption: mast cells

**DOI:** 10.1590/2177-6709.24.2.020-031.oin

**Published:** 2019

**Authors:** Solange de Oliveira Braga Franzolin, Maria Inês Moura Campos Pardini, Leda A. Francischone, Elenice Deffune, Alberto Consolaro

**Affiliations:** 1 Universidade Nove de Julho, Faculdade de Medicina (Bauru/SP, Brazil).; 2 Universidade Estadual Paulista, Faculdade de Medicina (Botucatu/SP, Brazil).; 3 Private Practice (Bauru/SP, Brazil).; 4 Universidade de São Paulo, Faculdade de Odontologia de Bauru (Bauru/SP, Brazil).; 5 Universidade de São Paulo, Faculdade de Odontologia de Ribeirão Preto, Pós-Graduação em Odontopediatria (Ribeirão Preto/SP, Brazil).

**Keywords:** Tooth eruption, Dental follicle, Mast cells, Odontogenesis

## Abstract

This study contributes to the understanding of the mechanisms associated with signs and symptoms of tooth eruption, by investigating the presence of mast cells in pericoronal tissues during the intraosseous (Group 1) and submucosal (Group 2) phases of eruption. We compared findings for these two groups with each other and with those for the oral mucosa (Group 3). In each group, 14 specimens were analyzed microscopically after hematoxylin and eosin staining and immunohistochemical analysis of c-Kit and tryptase expression. Results revealed that the number and density of mast cells is different in follicular tissues according to the eruption phase, which may mean that: 1) masticatory trauma of the oral mucosa and dental follicles in the submucosa may explain why reduced enamel epithelium exposes enamel to the cells of the connective tissue; 2) exposure of antigenic enamel proteins might correspond to the release of sequestered antigens, which may lead to the interaction of IgE and a greater number of mast cells in the region; and 3) the consequent degranulation and the local release of mediators, such as histamine, leukotrienes, prostaglandins, proteases, cytokines and growth factors, contribute to the understanding of signs and symptoms associated with tooth eruption.

## Resumo

Para contribuir com a compreensão dos mecanismos relacionados à sintomatologia e aos sinais associados à erupção dentária, investigou-se a presença de mastócitos nos tecidos pericoronários na fase intraóssea (Grupo 1) e submucosa (Grupo 2), comparando-os entre si e com a mucosa bucal (Grupo 3). Em cada grupo, 14 espécimes foram analisados microscopicamente em cortes corados com hematoxilina e eosina, e imunocitoquimicamente marcados com Ckit e Triptase. Pelos resultados obtidos, concluiu-se que a quantidade/densidade dos mastócitos é diferente nos tecidos foliculares de acordo com a fase de erupção, o que permite inferir que: 1) O traumatismo decorrente da mastigação sobre o conjunto “mucosa bucal com o folículo pericoronário na submucosa” pode explicar porque o epitélio reduzido exporia o esmalte às células do tecido conjuntivo; 2) A exposição das proteínas do esmalte com propriedades antigênicas corresponderia à liberação de antígenos sequestrados que levariam à interação de IgE e mastócitos em número aumentado na região; e 3) A consequente degranulação e liberação de mediadores no local, como histamina, leucotrienes, prostaglandinas, proteases, citocinas e fatores de crescimento, contribuem para a compreensão dos sinais e sintomatologia atribuídos à erupção dentária.

## INTRODUCTION

Tooth eruption may be divided into three phases according to bony crypt movements: 1) pre-eruptive; 2) eruptive, or pre-functional; and 3) post-eruptive, or functional.^1^

Fully formed enamel is covered with reduced enamel epithelium. Together with the capsular connective tissue that nourishes it, it forms the dental follicle, or dental sac, which has a fundamental role in tooth eruption, confirmed by the fact that its removal interrupts this process.^2^

Dental follicles have a high concentration of chemical mediators of osteoclasis,^3^ ^-^ ^6^ such as:

a) Prostaglandins;
b) Epidermal growth factor (EGF);
c) Interleukin-1 (IL-1);
d) Bone morphogenetic protein 4 (BMP-4);
e) Colony-stimulating factor 1 (CSF-1);
f) Transforming growth factor (TGF-β).

They act as a cascade of molecular signs, beginning with EGF or TGF-β1, which increases the genic expression of IL-1α in the stellate reticulum. IL-1α activates CSF-1, which takes part in the influx of monocytes in the pericoronal tissues, which differentiate into the osteoclasts necessary for tooth eruption.^2^ ^,^ ^7^

Deciduous tooth eruption has already been erroneously associated with several health disorders, such as sleep disruption, ear and cheek itching, primary herpetic gingivostomatitis, cough, croup, bronchitis, diarrhea, fever, convulsions and even death^8^. McDonald and Avery^9^ described tooth eruption as a physiological process, which does not justify its association with fever and systemic disorders. Fever and respiratory tract infections in this period of life may happen at the same time as eruption, but are not associated with it. 

Inflammation of gingival tissues before the full emergence of the crown may cause transient pain for a few days. Other signs and symptoms believed to be associated with this phase of eruption are irritability, sleep disruption, gingival inflammation, salivation, diminished appetite, diarrhea, intraoral ulcers, temperature increase, need to bite objects, itching and earache.^8^

Pierce et al.^10^ conducted an experimental study using a murine model and immunohistochemistry, and found IgE in the follicular tissues during the enamel maturation phase, which suggests that it is a consequence of exposure of enamel matrix proteins to immunocompetent cells in the extrafollicular connective tissue. 

This local hypersensitivity reaction may explain the frequent clinical signs found during tooth eruption. The tissues that surround the erupting tooth accumulate inflammatory cells, especially lymphocytes^11^ and increased numbers of mast cells,^12^ evidencing that the enamel matrix proteins are sequestered and antigenic.^13^ ^-^ ^17^

Mast cells were first described in 1877 by Ehrlich. They derive from pluripotential cells of the bone marrow and reside in connective tissue, predominantly near blood vessels, nerves and subepithelial tissues. A mature mast cell is relatively large, fusiform, polygonal or oval, measuring 15 to 20 µm, with a cytoplasm slightly eosinophilic and full of granules loaded with mediators. The nucleus is basophilic, slightly eccentric and relatively large, measuring 4 to 7 µm in diameter and having multiple chromatin agregates.^18^

The number of mast cells in human skin ranges from 5,120 to 9,472 per cubic millimeter^18^, but they are found in innumerable other sites, such as lungs, bronchi, nose, adenoid glands, lymph nodes, intestines, liver, iris, heart^18^ ^,^ ^19^ and oral tissues.^20^ ^-^ ^22^ After activation, they release their granules, rich in chemical mediators, such as histamine, proteoglycans, proteases, bFGF, NO, MMPs, acid phosphatase and cytokines, including interferons (IFN), TNF-α and interleukins (IL), as well as products derived from arachidonic acid, such as prostaglandins and leukotrienes.

Because of the great variety of active pharmacological mediators in its granules, mast cells have a fundamental role in both hypersensitivity reactions and inflammation. Mast cells significantly affect independent or IgR-dependent responses, extending its potential as pro-inflammatory effector cells to the regulating components of the immune system and contributing to the development and amplification of specific and unspecific inflammatory responses

The study conducted by Pierce et al^10^ suggests the question about whether mast cells may be present in dental follicles, in the intraosseous phase of tooth eruption, in similar distribution and number to those of the oral mucosa and of the dental follicles during the submucosal phase. The answer to this question may help clarify some anatomic and physiological aspects of tooth eruption and its clinical signs and symptoms. 

## MATERIAL AND METHODS

### Sample

**Figure 1:**
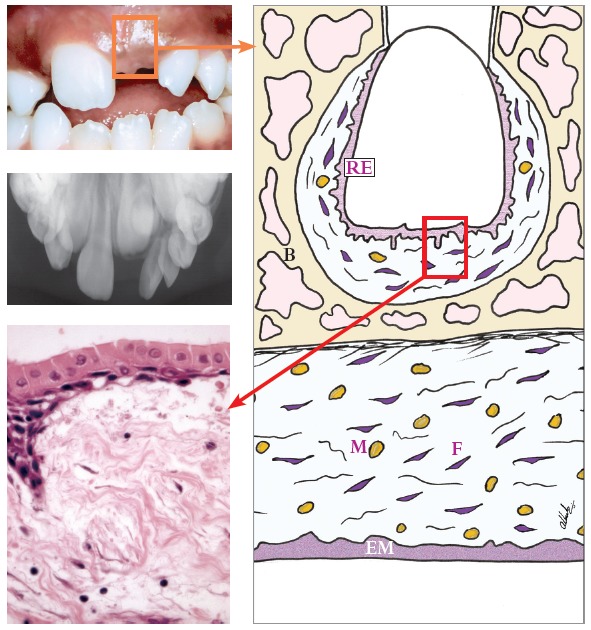
Dental follicle in intraosseous phase of eruption (Group 1) shows minimal number of mast cells (M - brownish circles). In oral mucosa, mast cells are sparse, diffusely and homogeneously distributed (RE = reduced enamel epithelium). F = fibroblasts; B = bone; EM = epithelium of the oral mucosa (HE; 40X).

**Figure 2:**
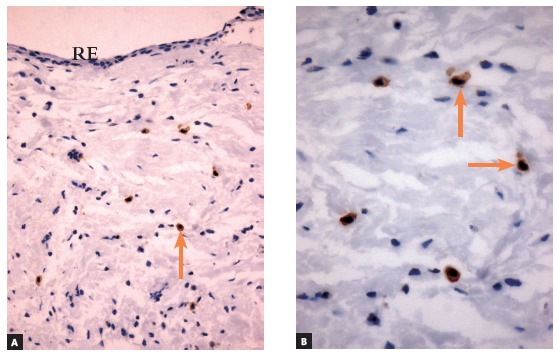
Immunolabeling with c-Kit (CD117) in dental follicle of tooth in intraosseous phase of eruption (Group 1), which had a minimal number of mast cells (arrows) (RE = reduced enamel epithelium) (A = 25X, B = 40X).

**Figure 3:**
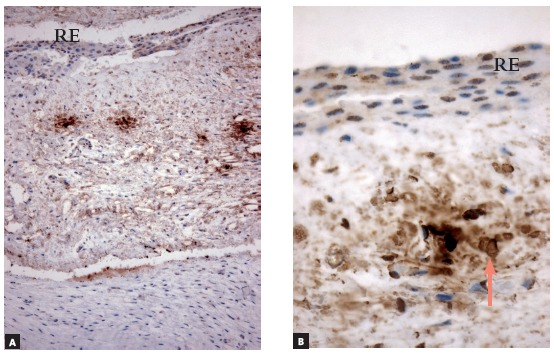
Antitryptase immunolabeling of dental follicle of tooth in intraosseous phase of eruption (Group 1), which had a minimal number of mast cells (arrows) (RE = reduced enamel epithelium) (A = 25X, B = 40X).

The tissue specimens examined in this study were collected from 42 healthy patients and divided into three groups: 

» Group 1 - dental follicles in the intraosseous phase of eruption: 14 surgical specimens of patients (3 men, 11 women; ages: 15 to 21 years) with an indication for extraction of unerupted intraosseous tooth. After osteotomy, the dental follicle was carefully removed before extraction (Figs 1 to 3).

**Figure 4:**
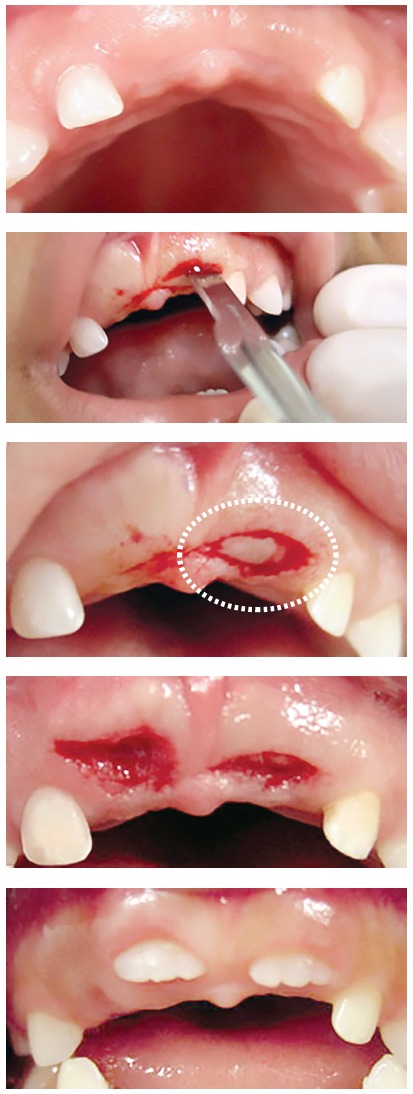
Harvesting of specimen for Group 2, composed of dental follicle of teeth in submucosal phase of eruption. Elliptical fragment (circle) and surgical bed during gingivectomy.

**Figure 5:**
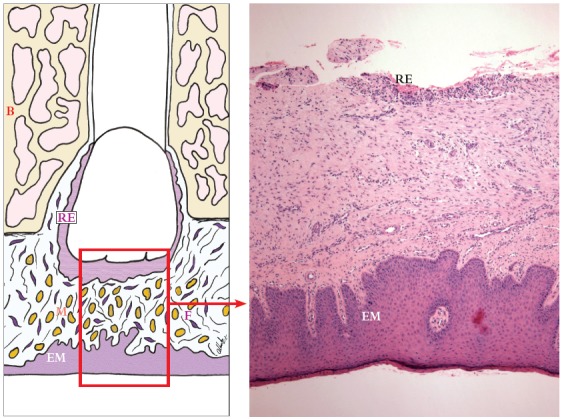
Dental follicle of teeth in submucosal phase of eruption (Group 2), which had a larger number of mast cells (M, brownish circles) (RE = reduced enamel epithelium); F = fibroblasts; B = bone; EM = epithelium of the oral mucosa (HE; 10X).

**Figure 6:**
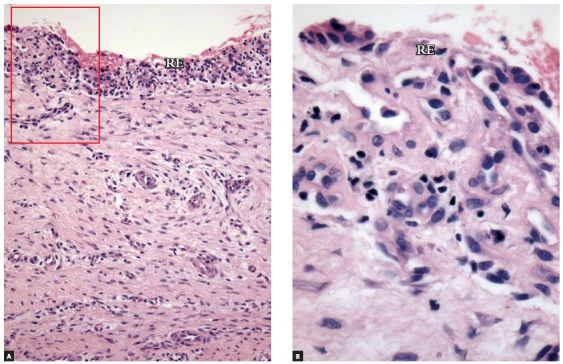
Dental follicle of teeth in submucosal phase of eruption (Group 2), which had ulcers and enamel exposure and was disorganized (RE = reduced enamel epithelium) (HE; A = 25X, B = 40X).

**Figure 7:**
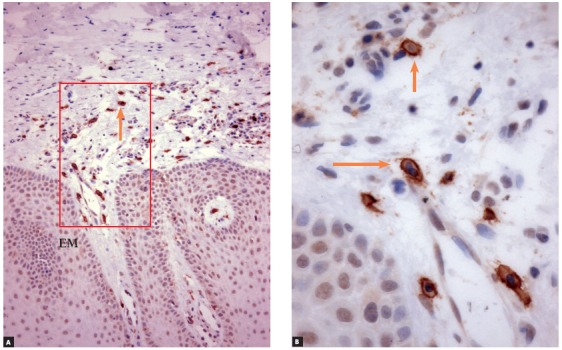
c-Kit (CD117) immunolabeling of dental follicle of tooth in submucosal phase of eruption (Group 2), which had a large number of mast cells (arrows) (EM = epithelium of the oral mucosa) (A = 25X, B = 40X).

**Figure 8:**
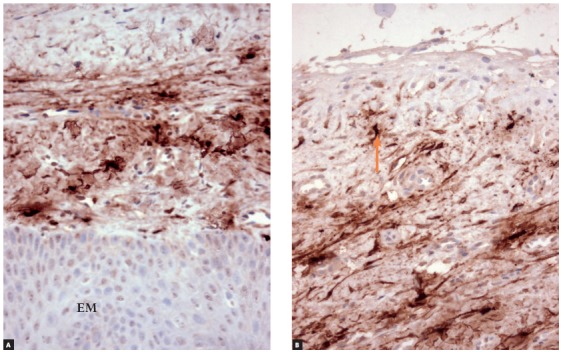
Antitryptase immunolabeling of dental follicle of tooth in submucosal phase of eruption (Group 2), which had a few mast cells (arrows) (EM = epithelium of the oral mucosa) (A = 25X, B = 40X).

» Group 2 - dental follicles in the submucosal phase of eruption: 14 surgical specimens of patients (8 men, 6 women; ages: 6 to 19 years) with an indication for gingivectomy. The gingiva and part of the dental follicle were removed (Figs 4 to 8).

**Figure 9:**
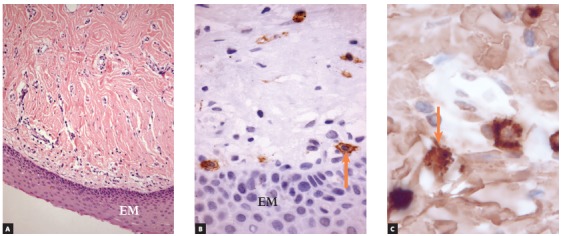
Oral mucosa (Group 3) with well distributed mast cells (arrows) in submucosa, but in smaller number than in Group 2, which was composed of follicles of teeth under eruption in submucosal phase (EM = oral mucosa epithelium) (A = HE; 25X; B = c-Kit (CD117), 40X; C = antitryptase, 100X).

» Group 3 - oral mucosa: 14 surgical specimens of patients (6 men, 8 women; age: 14 to 82 years) with an indication for removal of inflammatory fibrous hyperplasia. The oral mucosa of the specimen margins was free of any pathological changes (Fig 9). 

All the specimens were fixed in 10% formalin, embedded in paraffin and sequentially sectioned at a thickness of 4 µm. Tissue sections were mounted on slides to be stained and immunolabeled. 

### Microscopic analysis 

The slides were stained with hematoxylin and eosin (HE) and photographed using light microscopy (Olympus CH2). The criteria for analyses included the morphological aspects of the lining epithelium of the connective tissue and of the odontogenic epithelium of the dental follicle. 

### Immunohistochemical analysis

Tissue sections underwent two types of immunolabeling:

» CD117 or c-Kit (polyclonal; Dako Inc., Carpinteria, CA): transmembrane protein that belongs to the class III receptor of tyrosine-kinase family. The natural ligand for CD117 has been called mast cell growth factor. CD117 is found in progenitor and precursor cells of all hematopoietic lineage, but not in mature hematopoietic cells, except for mast cells.
» Tryptase (polyclonal; Chemicon International, Inc., Billerica, MA): family of trypsin-type neutral serum proteases, predominantly found in mast cells. As active enzymes, tryptases in mast cells are noncovalent tetramers with 132 kDa. The determination of mast cells using tryptases has been useful in the identification of focal or diffuse infiltrates rich in mast cells, as well as of atypical mast cells with little granulation or non-metachromatic.

Four-µm sections were mounted on slides and fixed at 70^o^ C for 12 hours. Samples were deparaffinized in four 5-min xylene baths, the sections were hydrated in PBS for five minutes, and endogenous peroxidase was blocked using 3% H_2_O_2_ (hydrogen peroxide) for five minutes. Antigen retrieval was performed in a steamer, in a pre-heated solution of 2.1% citric acid, pH 6, for 40 min at a temperature of up to 100o C. The Dako K4001 Envision System HRP labeled polymer antimouse was used for detection. 

The slides were incubated in a refrigerator at 4^o^ C overnight. When removed from the refrigerator, they were rinsed in PBS, and a 0.1% diaminobenzidine (DAB) chromogen was used for five minutes. The sections were washed in distilled water and counterstained with Mayer’s hematoxylin.

The number of mast cells was determined as the mean number of cells detected in ten different microscopic fields for each specimen, using a 40X objective lens, at 140 fields for each group and for each immunolabel^23^. Therefore, 280 fields were used for each group.

### Statistical analysis

Results are reported as mean number of immunolabeled cells in the microscopic fields. The Student*t* test was used to compare the two immunolabeling methods (c-Kit and tryptase) in the same group; and ANOVA was used for the comparisons between the three groups. The level of significance was set at 5% for all the analyses.

## RESULTS

### Microscopic analysis 

#### Group 1 - dental follicles in the intraosseous phase of eruption 

Dental follicles had reduced enamel epithelium with four to five layers and projections that invaded the connective tissue (Fig 3A). On the surface, there was a continuous cuboidal cell layer composed of reduced ameloblasts (Fig 3B). These superficial cells had eosinophilic cytoplasm with distinct borders, whereas their nuclei had moderately condensed chromatin. In the underlying layers, cells were spherical, and their nuclei were strongly stained. Reduced enamel epithelium was not continuous along the surface of the follicular connective tissue and had areas where structure and organization were intact (Fig. 1).

The follicle in eleven specimens had reduced epithelium of the enamel organ. The other three specimens had a stratified squamous epithelium with three to five cell layers. Follicular connective tissue was loose in some cases, mainly in the subepithelial and perivascular spaces; in other cases, it was fibrous. Interspersed islands and cords of odontogenic epithelium, remnants of the dental lamina, were regularly observed. No areas of inflammatory infiltrate or exudate were found in teeth in the intraosseous phase of eruption.

#### » Group 2 - dental follicles in the submucosal phase of eruption 

Dental follicles had an overlying oral mucosa, lamina propria and submucosa that contained follicular connective tissue with fragments of reduced enamel epithelium. In the oral mucosa, stratified squamous epithelium had an average of 15 to 30 cell layers, with discreet to moderate hyperplasia. In the lamina propria and submucosa, there were sporadic polymorphonuclear and mononuclear leukocytes. There were rare and scattered islands of odontogenic epithelium. The number and aspect of blood vessels were normal (Figs 5 and 6).

On the internal surface of the tissue fragment of dental follicle, the reduced enamel epithelium was not continuous. In four specimens, the internal lining was composed of reduced enamel epithelium; in ten, of stratified squamous epithelium. The fibrous connective tissue of the oral mucosa was continuous with the follicular connective tissue, with no signs of clear borders between them. The connective tissue near the follicular epithelium was looser and less collagenous.

#### » Group 3 - oral mucosa

Specimens in this group had stratified epithelium with 15 to 30 layers, and lamina propria was free of inflammation. Epithelium was parakeratinized and, in some areas, moderately hyperplastic. In the oral submucosa, fibrous connective tissue had sporadic leukocytes, but their morphology did not indicate inflammation (Fig. 9).

### Immunohistochemical analysis

» Group 1 - Immunolabeling of follicular tissues in the intraosseous phase using both c-Kit (Fig. 2) and tryptase (Fig. 3) revealed a small number of mast cells, with some specimens with no mast cells at all. In this group, mean number was 0.5 cells, with a standard deviation of 0.63.
» Group 2 - Immunolabeling of dental follicles in the submucosal phase of eruption using c-Kit and tryptase revealed a marked presence of mast cells in the submucosal connective tissue and in the dental follicle. Mean number of mast cells in the microscopic fields was 10.96, with a standard deviation of 2.67 cells (Figs 7 and 8).
» Group 3 - In the oral mucosa, mast cells immunolabeled using c-Kit and tryptase were scattered. Mean number of mast cells per microscopic field was 4.4, with a standard deviation of 1.63 (Fig 9).

The number of mast cells (mean and standard deviation) in the three groups is shown in Table 1. There were no significant statistic differences between immunolabeling methods (*p*> 0.05). The evaluation of the same immunolabeling in the different groups, considering Group 1 and Group 2, Group 1 and Group 3, and Group 2 and Group 3, revealed a statistically significant difference (*p*< 0.05) in all comparisons (Tab. 2). 

**Figure 10:**
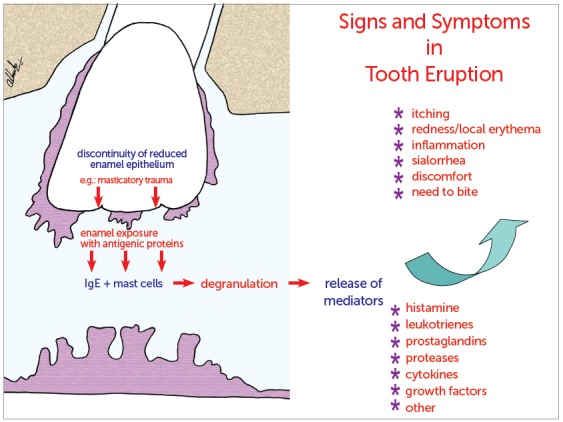
Schematic representation of mechanism suggested to explain signs and symptoms associated with tooth eruption.

**Table 1:** Evaluation and comparison of mean number of mast cells in the connective tissue of dental follicles (G1 and G2) and oral submucosa (G3), according to the techniques used (c-Kit and tryptase) for each group.

immunolabeling technique / Groups	c-Kit	Tryptase	P-value*
	Mean ± SD	Mean ± SD	
G1	0.51 ± 0.62	0.84 ± 0.97	0.28^**^
G2	10.96 ± 2.67	11.50 ± 2.43	0.59^**^
G3	4.40 ± 1.63	4.91 ± 1.23	0.36^**^

* Student t test; values below 0.05 indicate statistically significant differences. ** no statistically significant differences.

**Table 2:** Evaluation and comparison of groups G1, G2 and G3 to each other, according to the immunolabeling used (c-Kit and tryptase).

immunolabeling	Groups			P-value*
	G1	G2	G3	
c-Kit	0.51	10.96	4.40	< 0.0001^**^
Tryptase	0.84	11.50	4.91	< 0.0001^**^

* ANOVA; values below 0.05 indicate statistically significant differences.. ** no statistically significant differences.

## DISCUSSION

The presence of IgE in dental follicles in the phase after enamel is secreted may confirm that there is a hypersensitivity reaction during tooth eruption, as reported by Pierce et al.^10^ The detection of IgE indicates the presence of mast cells, which adapt to specific sites and functions.^18^ ^,^ ^19^ They respond to immunological and inflammatory stimuli, but were not found in the tissues of the dental follicles in the intraosseous phase (x = 0.51 per microscopic field) (Table 1). The epithelium of the dental follicle, when intact and intraosseous, seems to protect the enamel by sequestering it from the action of these cells or of IgE. 

Group 2 represented the moment of tooth eruption, the submucosal phase, during which the epithelium of the dental follicle was no longer continuous, with areas where the enamel was exposed to the extrafollicular connective tissue. Immunolabeling of these follicles using c-Kit and tryptase revealed a large number of mast cells. 

Pierce et al^10^ found IgE on the surface of ameloblasts in the post-secretory phase, suggesting that it is a result of the exposure of enamel matrix proteins to immunocompetent cells of the extrafollicular connective tissue, which confirms the present findings. Their study was conducted with a murine model, and the phases might be coincident, as they reported that IgE was clearly observed at 15 days of life, but was scarce at 5 days. Findings were associated with the immunoresponse and signs and symptoms during the emergence of teeth in the oral cavity.^10^ ^-^ ^13^

The analysis of mast cells revealed that the three groups had distinct results, with differences that were not markedly significant. Group 1 had few mast cells in only a few specimens. These results could not be compared with others in the literature, because the present search did not retrieve any studies with similar methods to detect mast cells in pericoronal tissues in this phase. The intact epithelium of the dental follicle probably protects the enamel from the action of the cells in the connective tissue, associated with immunopathological reactions.

In contrast, Group 2 had a larger number of immunolabeled mast cells. The ruptured epithelium of the dental follicle and the exposure of enamel to the connective tissue may attract more mast cells to the region, because enamel has proteins that may act as sequestered antigens.^10^ ^,^ ^13^

In normal oral submucosa (Group 3), the number of mast cells was much smaller than in the connective tissue of the dental follicles during the submucosal phase. Masticatory trauma of oral mucosa and dental follicles in the submucosa may explain why reduced enamel epithelium exposes enamel to the cells of the connective tissue. These traumas may promote the discontinuity of the reduced enamel epithelium, frequently seen in Group 2.

Exposure of antigenic enamel proteins might correspond to the release of sequestered antigens.^13^ Such exposure may hypothetically lead to the interaction between IgE and mast cells at a larger number in the region, and to the consequent degranulation and local release of mediators, such as histamine, leukotrienes, prostaglandins, proteases, cytokines and growth factors. This process helps the understanding, at least partially, of the occurrence of signs and symptoms associated with tooth eruption, such as itching, inflammation, local redness and increased salivation. 

## Conclusions

The results of this study suggest that:

1. The amount/density of mast cell is different in follicular tissues according the eruption phase.
2. During the intraosseous phase of eruption, immunohistochemical analyses do not reveal any marked presence of mast cells in follicular connective tissues.
3. In the submucosal phase, the number of mast cells is significantly higher than in the intraosseous phase of tooth eruption, and in the submucosa of the normal oral mucosa not associated with teeth.

These results suggest that:

1. Trauma due to mastication on the set of oral mucosa and dental follicles in the submucosa may explain why reduced enamel epithelium exposes enamel to the cells of the connective tissue.
2. Exposure of antigenic enamel proteins might correspond to the release of sequestered antigens that would lead to the interaction of IgE and the greater number of mast cells in the region.
3. The consequent degranulation and the local release of mediators, such as histamine, leukotrienes, prostaglandins, proteases, cytokines and growth factors, contribute to the understanding of signs and symptoms assigned to tooth eruption, such as itching, inflammation, local redness and sialorrhea.
